# Absolute and Functional Iron Deficiency Anemia among Different Tumors in Cancer Patients in South Part of Iran, 2014

**Published:** 2017-07-01

**Authors:** Seyed Mehdi Hashemi, Mohammad Ali Mashhadi, Mehdi Mohammadi, Maryam Ebrahimi, Abolghasem Allahyari

**Affiliations:** 1 Department of Internal Medicine, School of Medicine, Ali-Ebne-Abitaleb Hospital Complex, Zahedan University of Medical Sciences, Zahedan, Iran; 2 Department of Statistics, School of Health Care, Zahedan University of Medical Sciences, Zahedan, Iran; 3 Department of Internal Medicine, School of Medicine, Mashhad University of Medical Sciences, Mashhad, Iran

**Keywords:** Iron deficiency anemia, Cancer, Hematologic malignancies, Chemotherapy

## Abstract

**Background: **Anemia is a common problem in cancer patients. This study aimed to investigate the frequency rate of absolute and functional iron deficiency anemia among different tumors and its distribution in different stages of cancer in solid tumors.

**Materials and Methods:** This study was performed on 597 patients with cancer referred to Ali-Ebne-Abitaleb Hospital in Zahedan. Laboratory tests included serum iron, transferrin saturation, C-reactive protein (CRP), erythrocyte sedimentation rate (ESR) and complete blood count (CBC). The malignancy type and stages were recorded. Data were analysed using SPSS statistics software (Ver.19).

**Results: **Four hundred and fifty-seven patients (76.5 %) diagnosed with solid tumors and 140 (23.5%) suffered from hematologic malignancies. Among patients with solid tumors, functional iron deficiency had the highest rate (300 patients had anemia and 243 (53.2%) of whom were functionally iron deficient), but in hematologic malignancies most of patients had not iron deficiency (66 patients had not iron deficiency against 12 patients had absolute iron deficiency and 62 patients had functional iron deficiency anemia) (P-value=0.021). No significant differences were observed among the various stages of cancers in terms of degrees of iron deficiency (P>0.05).

**Conclusion:** The results of the study showed that solid tumors had a higher rate of absolute and functional iron deficiency anemia, compared to hematologic malignancies. But there was no difference between the different stages of the disease.

## Introduction

 Anemia is a common problem in cancer patients^1^.It is observed in 50% of patients with solid tumors and 70-60% of patients with hematologic malignancies^2^.

There are various factors that play a role in cancer-related anemia such as the type of cancer, bleeding tumor, duration and stage of disease, bone metastasis, malnutrition, infection, cytokine secretion and cancer treatment^3^^, ^^4^.

The treatment method including surgery, radiation and chemotherapy are effectively involved in incidence and severity of anemia^5^. Tissue hypoxia induced by prolonged chronic anemia can induce severe damages to cardiovascular system, immune system, respiratory system, renal system and central nervous system^6^.

Anemia plays an important role in prognosis and survival of patients with cancer.^7^ Anemia overshadows the effectiveness of cancer treatment by increasing tumor cell resistance to therapy and reducing patient’s survival^8^.

In a study by Shen et al., 10-year survival in gastric cancer patients with anemia was 1.76%, while it was 5.83 % in patients without anemia^9^.

Anemia is accompanied by low hemoglobin levels and clinical symptoms such as social isolation, depression and cognitive impairment, dyspnea, tachycardia, dizziness, cardiac hypertrophy, reduced temperature and pale skin, loss of appetite and digestive disorders^6^.

Functional iron deficiency (FID) is a state in which there is insufficient iron incorporation into erythroid precursors in the face of apparently adequate body iron stores^10^. In this condition, partial block in iron transport to the erythroid marrow is seen in subjects with infectious, inﬂammatory and malignant diseases, and it is an important component of the anemia of chronic disease (ACD). Laboratory findings in such situations have indicated iron deficiency in spite of the enough iron content in body. Assessment of body iron stores is useful for diagnosis and treatment follow- up^10^. Evaluation of iron status is essential for the management of patients with FID. SI, TIBC and transferrin saturation are other variables that may be useful in the diagnosis of FID. The main problem in anemia of chronic disease and anemia of malignancies is failure to deliver enough iron to erythroid cells^10^.

 Ludwig et al. (2013) studied the prevalence of iron deficiency across different tumors, and they evaluated 1528 patients with cancer; of whom 1053 had solid tumors, and 475 had hematologic malignancies. Transferrin saturation (TSAT) less than 20% was noted in 42.6% of patients, moreover, 33.0% of patients were anemic. The highest ID rates were observed in pancreatic (63.2%), colorectal (51.9%) and lung cancers (50.7%), respectively. Furthermore, in patients with solid tumors, the prevalence of ID was found to be correlated with cancer stage and the response to treatment^11^.

In a research conducted by Zeighami Mohammedi (2011), the relationship between anemia and severity of fatigue and quality of life in cancer patients undergoing chemotherapy was studied. Prevalence of anemia in 121 cancer patients undergoing chemotherapy was 63.6%. In addition, 57% of patients had mild anemia and 6.4% had moderate to severe anemia^12^.

Today, the survival of patients is not the only consideration and individuals would like to live with optimal quality, so identification of factors improving the quality of life seems to be necessary. As anemia has been proposed as one of the common side effects along with cancers and cancer treatment, this study was designed to determine the prevalence of absolute and functional iron deficiency anemia among a variety of tumors and its relationship with the patient's functional status in Ali-Ebne-Abitaleb Hospital, Zahedan, Iran (2004).

## MATERIALS AND METHODS

 To conduct this descriptive-analytical research, 597 cancer patients referred to Ali-Ebne-Abitaleb Hospital in Zahedan, Iran were recruited through a convenience sampling method. The inclusion criteria for this study were: diagnosis of cancer, having a treatment of less than 4 weeks and being over 18 years of age. Patients who had the history of blood transfusion over the past 3 months, blood donation during the last 3 months, consumption of supplements containing iron compounds and individuals with incomplete information were excluded.

After obtaining informed consent, demographic features, cancer stage, and cancer type were recorded on a survey form especially designed for the purpose. Furthermore, laboratory assessment of iron stores including ferritin, serum iron, transferrin saturation, C-reactive protein (CRP), erythrocyte sedimentation rate (ESR) and complete blood count (CBC) was undertaken . This information included patients' anemia status based on hemoglobin level as values<12 g/dL in women and <13 g/dL in men were defined as anemia according to WHO. Absolute iron deficiency was defined as transferrin saturation (TSAT) less than 20% with ferritin levels below 40 micrograms per litter, and functional iron deficiency was defined as TSAT less than 20% with ferritin levels above 40 micrograms per litter. Also, the collected information such as type of tumor and tumor stage (metastatic / non metastatic for solid tumors - acute / chronic for leukemia - I to IV for lymphoma) were recorded on the forms. After collecting and completing information about patients, the data was entered to statistical software SPSS19 and analyzed through descriptive statistical techniques (Chi-square). The statistics at p-value<0.05 was considered significant. 

## Results

 Five hundred and ninety-seven patients with mean age of 52.56 ± 15.34 years were included in the study; of whom 285 (47.7%) were men and 312 (52.3%) were women. One hundred and forty (23.5%) of cases were suffering from hematologic malignancies, and 457 (76.5%) were suffering fromsolid tumors. In the present study, the most common sites of malignancies were within the gastrointestinal tract (n=122, 20.4%) and breast (n = 100, 16.8%) (Table 1). 

**Table 1:** The frequency of malignancy in the study

**Disease**		**Frequency**	**Percent**
Solid Tumor	Pancreas	7 patients	1.2 %
	Colorectal	63	10.6 %
	Lung	35	5.9 %
	Esophagus and GI	122	20.4 %
	Kidney and urinary tract	37	6.2 %
	Breast	100	16.8 %
	Obstetrics and Gynecology	24	4.0 %
	Testis	5	0.8 %
	Others	64	10.7 %
	Total	457	76.5 %
Hematological malignancies	Lymphoma	87 patients	14.6 %
	Leukemia	44	7.4 %
	Myeloma	9	1.5 %
	Total	140	23.5 %
Total		597 Patients	100 %

There was no statistically significant difference in the mean age of patients with different iron deficiency (P=468/0) (Table 2). 

**Table 2:** The mean age of the patients according to the type of iron deficiency

**Iron deficiency**	**Age (Mean ± SD)**	**P-value**
Absolute	54.14 ± 14.1 years	0.486
Functional	52.83 ± 15.39 years	
None	51.7 ± 15.65 years	
Total	52.56 ± 15.34 years	One-way ANOVA

In the distribution of iron deficiency, no statistically significant difference was observed between the different age groups, regardless of the type of disease (P-value=0.206) (Table 3), and this was also true for each type of solid and hematological tumors (P-value=0.11 and P-value=0.929, respectively). No statistically significant difference was observed between different genders in terms of distribution of iron deficiency (P=0.296) (Table 4). 

**Table 3:** The prevalence of iron deficiency by age group

**Age group**	**Iron deficiency**			**P-value** *
	**Absolute ** **(Frequency, ** **percent)**	**Functional ** **(Frequency, ** **percent)**	**Non ** **(Frequency, ** **percent)**	
Less than 40 years	11 (15.9 %)	77 (25.9%)	61 (27.4 %)	0.206
40 – 60 years	37 (53.6 %)	125 (41.0 %)	86 (38.6 %)	
Over 60 years	21 (30.4 %)	103 (33.8 %)	76 (34.1%)	
Total	69 (100%)	305 (100%)	223 (100%)	

* Chi-square test

**Table 4:** The prevalence of iron deficiency in terms of gender

**Gender**	**Iron deficiency**			**P-value** *
	**Absolute ** **(Frequency, ** **percent)**	**Functional ** **(Frequency, ** **percent)**	**Non ** **(Frequency, ** **percent)**	
Male	27 (39.1 %)	151 (49.5%)	107 (48.0 %)	0.296
Female	42 (60.9 %)	154 (50.5 %)	116 (52.0 %)	
Total	69 (100 %)	305 (100 %)	00 %)	

* Chi-square test

Distribution of different degrees of iron deficiency depending on the type of disease (solid tumor or hematologic malignancy) is shown in Table 5. 

**Table 5:** The prevalence of iron deficiency in terms of disease

**Iron deficiency**	**Disease**		**P-value** *
	**Solid tumor**	**Hematological ** **malignancy**	
Absolute	57 (12.5 %)	12 (8.5 %)	0.021
Functional	243 (53.2 %)	62 (44.3 %)	
None	157 (34.4 %)	66 (47.1 %)	
Total	457(100 %)	140 (100 %)	

* Chi-square test

According to this Table, patients with solid tomors had the highest incidence of functional iron deficiency (2.53%), while most patients with hematologic malignancies did not have iron deficiency (1.47%). There was a statistically significant difference in the frequency of iron deficiency between the two types of malignancy according to Chi-square test results (P-value=0.021). 

In terms of frequency distribution of various degrees of iron deficiency depending on the stage of the disease, a significant difference was observed between acute and chronic leukemia (005/0 = P), but regarding the other hematologic malignancies and also solid tumors there was no significant difference between the various stages of the disease in terms of iron deficiency. (0.05 <P-value) (Table 6).

**Table 6:** The prevalence of iron deficiency in terms of disease stage

**Type of Tumor**	**Disease stage**		**Iron deficiency**			**P-value** *
			**Absolute**	**Functional**	**Non**	
Solid Tumors	Metastatic		23 (40.4%)	116 (47.7%)	80 (51.0%)	0.388
	Non-metastatic		34 (59.6%)	127 (52.3%)	77 (49.0%)	
	Total		57 (100%)	243 (100%)	157 (100%)	
Hematological malignancies	Lymphoma	I	3 (42.9%)	7 (20.6%)	7 (15.2%)	0.488
		II	3 (42.9%)	13 (38.2%)	17 (37.0%)	
		III	1 (14.3%)	10 (29.4%)	19 (41.3%)	
		IV	0 (0%)	4 (11.8%)	3 (6.5%)	
		Total	7 (100%)	34 (100%)	46 (100%)	
	Leukemia	Acute	0 (0%)	16 (80.0%)	9 (45.0%)	0.005
		Chronic	4 (100%)	4 (20.0%)	11 (55.0%)	
		Total	4 (100%)	20 (100%)	20 (100%)	
	Myeloma		1 (100%)	8 (100%)	(0%)	

* Chi-square test

The mean ESR level was different between patients with different degrees of iron deficiency (0.001> P-value). ESR was in the highest average level (48.62 ± 27.79) in the patients with functional iron deficiency, but no significant difference was observed in the level of CRP between patients with various degrees of iron deficiency (p-value=0.429) (Table 7).

**Table 7:** The average amount of ESR in terms of iron deficiency

**Iron deficiency**	**ESR (Mean ± SD)**	**P-value** *
Absolute	40.62 ± 68.58	˂ 0.001
Functional	48.62 ± 27.79	
Non	37.72 ± 26.79	

* Kruskal-Wallis Test

Most patients with varying degrees of iron deficiency had performance status of 1 (ECOG [Eastern Cooperative Oncology Group]), but, in general, statistically significant differences were observed in terms of patient’s performance status between different degrees of iron deficiency (ECOG) (P-value=0.01) (Table 8).

**Table 8:** The prevalence of iron deficiency in patients with performance

**Patients performance**	**Iron deficiency**			**P-value** *
	**Absolute**	**Functional**	**Non**	
0	14 (20.3%)	16 (5.2%)	27 (12.1%)	0.01
1	37 (53.6%)	173 (56.7%)	124 (55.6%)	
2	14 (20.3%)	101 (33.1%)	59 (26.5%)	
3	3 (4.3%)	11 (3.6%)	10 (4.5%)	
4	1 (1.4%)	4 (1.3%)	3 (1.3%)	
Total	69 (100%)	305 (100%)	223 (100%)	

* Chi-square test

## Discussion

 In the present study, 76.5% of patients (n=457) had Solid tumors and 23.5% (n=140) had hematologic malignancies. The frequency of absolute and functional iron deficiency varied among different tumors, and it was more functional iron deficiency in patients with solid tumors. The highest absolute iron deficiency was mostly observed in*menopausal* women with *breast cancer* due to low iron intake or their life style (n = 24, 34.8%) and functional iron deficiency was observed in patients (n=71, 23.3%) with gastrointestinal cancer (Table 9). 

**Table 9:** Most frequencies of Iron deficiency in each tumors

**Iron deficiency**	**Tumors**	
Absolute	Breast cancer	24 (34.8%)
	Others*	45 (65.2%)
	Total	69 (100%)
Functional	Gastrointestinal Tumors	71 (23.3%)
	Others*	234 (76.7%)
	Total	305 (100%)

* Other tumors facts were scattered and non-homogenous and do not included in the study.

The most frequent absolute and functional iron deficiency was found in patients with performance status levels (ECOG) of 1 and 2. The highest level of ESR was also observed in functional iron deficiency. 

Iron status was basically assessed using TSAT, which somewhat was influenced by inflammation. As ferritin is an acute-phase reactant^13^^, ^^14^and also due to inflammation associated with cancer, it will not present an accurate picture of iron deposits in this disease^15^. If ferritin (with the cut-off of fewer than 30 nanograms per milliliter) is used alone in the evaluation of iron deficiency, a large part of the patients with the functional iron deficiency will not be diagnosed. This shows the importance of TSAT as a biomarker for assessing iron in cancer patients, but previous studies have indicated that TSAT is often not used in assessment^18^^, ^^19^.

Due to lack of similar studies, our findings cannot be compared to prior studies, like the survey conducted by Kuvibidila et al. (2004) in which a low TSAT and a high TIBC in 34 men with prostate cancer were compared to the control group. TSAT was less than 16% in 31.6% of patients, while it was 8.6 % in the control group^19^. Even Robertson and Hutchinson (2009) reported iron deficiency in 9% of anemic cancer patients^16^.

In a study conducted by Beale et al. (2013), it was reported that 60% of 130 patients with colorectal cancer had iron deficiency. The study also showed a decrease in TSAT (42.3%) and ferritin levels (13.8%) among patients with colorectal cancer. Moreover, 69% of patients with low TSAT (less than 16%) had anemia^20^.

Ludwig et al. (2013) conducted a study and evaluated the prevalence of anemia *across different* solid *tumor* types and its association with iron deficiency anemia. Iron deficiency (transferrin saturation less than 20%) was found in 645 (42.6%)/ 1513 patients; of whom 500 (33.0%) had iron deficiency. Prevalence of iron deficiency was higher in pancreatic cancer (63.2%), colorectal cancer (51.9%) and lung cancer (81.9%). In 409 patients with iron deficiency, in addition to transferrin saturation, ferritin was also available at the same time, 335 patients (81.9%) had functional iron deficiency (20%> TSAT, 30 ng/ml≤ Ferritin) and 74 (18.1%) had absolute iron deficiency. The prevalence of iron deficiency in patients with solid tumors was related to the stage of the disease at the time of diagnosis, the patient's condition and performance status (ECOG) (p-value was 0.001, 0.001 and 0.005, respectively). This study showed that iron deficiency was frequently observed in malignancy and it has a close relationship with poor performance status (ECOG) and disease progression in these patients^11^.

Knight et al. (2004) in the United States conducted a study to assess the prevalence of anemia in cancer and the effect of anemia on survival and quality of life of patients. Most studies have reported that 30% to 90 % of cancer patients have anemia, depending on Hb level chosen to define it. For instance, when anemia was defined as haemoglobin level less than 9 gram per liter, 7% of patients with Hodgkin’s disease were found to be anemic. But when the haemoglobin level less than 11 grams per liter was considered as anemia, 86% of patients were found to be anemic. The prevalence of anemia also varies with the type of disease and its stage. Forty percent of patients with early-stage colon tumors and 80% of advanced-stage patients were anemic. Patients with anemia had poorer survival and tumor control than patients without anemia. Quality of life was associated positively with hemoglobin levels. There were no significant differences in the treatment of toxicity between anemic and non-anemic patients. As a result, the treatment of anemia can have a remarkable impact on survival and quality of life^1^.

In Poddar et al.’s case report (2011), the association between small intestine cancer and iron deficiency anemia was found in a 56 –year-old female patient. She was treated with chemotherapy and showed moderate response to treatment. Severe iron deficiency in this patient revealed the importance of examining the association between iron deficiency and cancer of the small intestine^21^.

In Zeighami Mohammadi et al. study (2011), the prevalence of anemia in cancer patients undergoing chemotherapy was 63.6%. In terms of the level of hemoglobin, there was a statistically significant difference in the severity of fatigue (0.001> P) and quality of life (0.003 = P). There was also a negative relationship between severity of fatigue and hemoglobin level as well as quality of life. As a result, anemia has a close relationship to fatigue and quality of life. During chemotherapy, a decrease in hemoglobin levels results in the decrease of physical, cognitive and social functions, severe fatigue and poor quality of life^12^.

In a study conducted by Zhen et al. (2012) in China, iron deficiency anemia as a predictor of long-term oncologic outcomes in patients with Stage II colon cancer was studied in two groups of T3N0M0 and T4N0M0. The results of the study showed that 147 (22.8%) of 644 patients had iron deficiency anemia. Prevalence of iron deficiency anemia showed no significant difference between the two groups (P=0.340). But in T4N0M0, the incidence of iron deficiency anemia increased by increasing tumor penetration. Iron deficiency anemia represents worse survival among patients with cancer T3N0M0, but it is not valid for patients with Stage III cancer. So, iron deficiency anemia is a long-term independent predictor for colorectal cancer in stage T3N0M0, but this fact is not true about T4N0M0 stage^22^.

Sideris et al. (2015) in Denmark studied 41 patients with colorectal cancer accompanied by iron deficiency anemia and BRAF V600E mutation. There was no significant correlation between Hb levels at presentation and disease stage. Patients with right-sided tumors were found to have lower Hb levels than patients with either left-sided colonic or rectal tumors. Hb levels were also significantly lower in patients with the BRAF V600E mutation. The findings of the study showed that BRAF V600E mutation might be associated with right-sided tumors, and subsequently related to unexplained iron-deficiency anemia (IDA) at presentation of disease iron-deficiency anemia (IDA)^23^.

The study showed that solid tumors have a higher percentage of absolute and functional iron deficiency than hematologic malignancies. Like hematologic malignancies, no significant difference was observed between metastatic and non-metastatic cases.

## CONCLUSION

 The results of this study indicate that there is an outbreak of iron-deficiency anemia, especially functional anemia in solid tumors. However, major studies on this issue was only limited to digestive cancers, nevertheless, the diversity of cancer patients in this study indicates the seriousness of the functional and absolute iron deficiency in patients with all types of solid tumors. Regarding the final outcome of the study and quality of life in cancer patients, more attention to this missing piece of puzzle is required to make the treatment of cancer more effective. As InPoddar et al.’s case report (2011) showed anemia has a close relationship to fatigue and quality of life. During chemotherapy, a decrease in hemoglobin levels results in the decrease of physical, cognitive and social functions, severe fatigue and poor quality of life^12^.
